# Comprehensive identification of a disulfidptosis-associated long non-coding RNA signature to predict the prognosis and treatment options in ovarian cancer

**DOI:** 10.3389/fendo.2024.1434705

**Published:** 2024-09-13

**Authors:** Shouze Liu, Rulan Jiang, Xinxin Wang, Qianqian Zhang, Shumei Li, Xiaoxue Sun, Yajun Feng, Feida Du, Pengtao Zheng, Yanpeng Tian, Zhongkang Li, Shikai Liu

**Affiliations:** ^1^ Department of Gynecology III, Cangzhou Central Hospital, Cangzhou, Hebei, China; ^2^ Department of Pain, Cangzhou Hospital of Integrated Traditional Chinese Medicine and Western Medicine (TCM-WM) Hebei, Cangzhou, Hebei, China; ^3^ Department of Gynecology and Obstetrics, Beijing Tsinghua Changgung Hospital, Beijing, China; ^4^ Department of Gynecology, The Second Hospital of Hebei Medical University, Shijiazhuang, Hebei, China; ^5^ Department of Obstetrics and Gynecology, The First Affiliated Hospital of Zhengzhou University, Zhengzhou, Henan, China

**Keywords:** disulfidptosis, lncRNA, ovarian cancer, signature, immunotherapy

## Abstract

**Purpose:**

Distinguished from cuproptosis and ferroptosis, disulfidptosis has been described as a newly discovered form of non-programmed cell death tightly associated with glucose metabolism. However, the prognostic profile of disulfidptosis-related lncRNAs (DRLRs) in ovarian cancer (OC) and their biological mechanisms need to be further elucidated.

**Materials and methods:**

First, we downloaded the profiles of RNA transcriptome, clinical information for OC patients from the TCGA database. Generated from Cox regression analysis, prognostic lncRNAs were utilized to identify the risk signature by least absolute shrinkage and selection operator analysis. Then, we explored the intimate correlations between disulfidptosis and lncRNAs. What’s more, we performed a series of systemic analyses to assess the robustness of the model and unravel its relationship with the immune microenvironment comprehensively.

**Results:**

We identified two DRLR clusters, in which OC patients with low-risk scores exhibited a favorable prognosis, up-regulated immune cell infiltrations and enhanced sensitivity to immunotherapy. Furthermore, validation of the signature by clinical features and Cox analysis demonstrated remarkable consistency, suggesting the universal applicability of our model. It’s worth noting that high-risk patients showed more positive responses to immune checkpoint inhibitors and potential chemotherapeutic drugs.

**Conclusion:**

Our findings provided valuable insights into DRLRs in OC for the first time, which indicated an excellent clinical value in the selection of management strategies, spreading brilliant horizons into individualized therapy.

## Introduction

As the most lethal gynecological tumor, ovarian cancer (OC) is usually characterized by difficult detection in the early stage and a lack of biomarkers, leading to a poor prognosis (1). OC has caused over 570,000 deaths worldwide annually according to the Global cancer statistics released in 2021 (2). Despite the past few years have witnessed dramatic advances in diagnosis and treatments of OC, the progression-free survival (PFS) remains short, in the range of 1 to 2 years (3), with an estimated 5-year survival rate of 30% (4). Therefore, it is urgently necessary to unearth efficient diagnostic and prognostic values to enhance survival outcomes for OC patients.

Disulfidptosis, a newly identified cell death manner under disulfide stress conditions, which is triggered by the overaccumulation of intracellular cystine, occurs typically in glucose-starved cells (5). In detail, the accumulation of a large number of disulfide molecules facilitates aberrant disulfide bonds between actin cytoskeletal proteins, resulting in actin filament contraction and detachment from the plasma membrane and ultimately causing the collapse of actin network and cell death. When cells with elevated SLC7A11 expression undergo glucose deprivation, disulfidptosis is initiated (5). Tumor cells rely on SLC7A11 for cysteine import to maintain redox equilibrium and ensure their survival (6). However, this dependence also highlights a critical vulnerability in SLC7A11 over-expressing cancer cells, as they need glucose to mitigate disulfide bond overload. Previous studies have indicated that the expression of SLC7A11 is significantly higher in ovarian cancer compared to normal tissues (7). Furthermore, *in vitro* studies have shown that the upregulation of SLC7A11 significantly enhances the migration and invasion of OC cells (8). Hopefully, targeting disulfidptosis may raise fresh opportunities for OC metabolic therapy in a regulated cell death way.

Non-coding RNAs (ncRNAs), defined as RNA molecules over 200 nucleotides in length, are a novel class of non-protein-coding transcripts, well known as microRNAs (miRNAs), circular RNAs (circRNAs) and long non-coding RNAs (lncRNAs) (9). Although they cannot be translated into proteins, they perform crucial functions in various physiological and pathological processes, including cancer (10). Among all ncRNAs, lncRNAs have been confirmed to have the highest potential of coding peptides, which are related to chromatin modifications, DNA transcription and the regulation of mRNA stability (9). Therefore, lncRNAs hold tremendous promise as candidate targets and brilliant biomarkers for cancer immunotherapies. It has been reported that disulfidptosis-associated lncRNAs (DRLRs) hold predictive prognostic significance in breast cancer (11, 12), cervical cancer (13), pancreatic cancer (14), colon cancer (15, 16) and lung adenocarcinoma (17, 18). In comparison, it is still in the dark ages about DRLRs implicated in OC.

In our work, we intended to develop a prognostic signature for OC patients, on the basis of DRLRs, which could possibly help to predict the overall survival (OS), explore differences in the immune microenvironment (TME), unravel the mechanisms underlying disulfidptosis and discover sensitive chemotherapeutic drugs for patients.

## Materials and methods

### Samples collection and data procession

We retrieved and downloaded the RNA transcriptome and clinical information of 379 OC patients from the TCGA-OV project (https://portal.gdc.cancer.gov/repository). These tumor samples were randomly assigned to two groups in a 1:1 ratio with excluding incomplete critical clinical information, leaving 187 patients in the training group and 187 patients in the test group. Moreover, we obtained the mRNA expression dataset of normal ovarian tissues (n=88) from the Genotype-Tissue Expression (GTEx) database as published. We manually compiled 30 validated disulfidptosis-related genes from updated literature (19–26), details of which were presented in **Supplementary Table 1**.

### Acquisition of DRLRs and construction of relevant prognostic index

First, we examined the correlation between lncRNAs and disulfidptosis-related genes by Pearson correlation coefficient (|R2| ≥ 0.4, *P* < 0.001) so as to determine candidate lncRNAs, which was visualized with a Sankey plot. At the same time, lncRNAs with prognostic clinical value were screened via univariate Cox regression analysis. After a two-step screening process, we speculated initial lncRNAs for subsequent investigation. Then, in the training cohort, least absolute and selection operator (LASSO) as well as multivariate Cox regression was conducted to consummate the preparation of core lncRNAs for predictive index establishment. We performed internal validation in the remaining testing samples and the whole TCGA group. Significantly, 1000-fold cross-validation was implemented to ascertain the optimal signature according to the penalization parameter (*P*<0.05). Finally, each patient’s risk score was calculated as per the following formula: risk score = lncRNA1 (coef * expression) + lncRNA2 (coef * expression…… + lncRNAn (coef * expression). As soon as the model was achieved, OC patients would be assigned to a high-risk (≥ median) group or a low-risk (< median) group on the basis of the median value calculated from the training cohort.

### Full-quality appraisal and validation of the model

To evaluate the general applicability of the DRLRs index, we drew OS and PFS curves by the Kaplan–Meier method with R packages “survminer” and “survival” in the training cohort, the testing cohort and the whole cohort, respectively. According to the risk type and corresponding survival time of each patient, survival status maps, risk curves and heatmap were plotted to determine if there was any difference in the distribution of the two groups. Through dimensionality reduction by PCA analysis, we visually demonstrated the spatial dispersion of patients with different risk clusters. Then, with the help of package “survival” in R, we conducted univariate and multivariate Cox regression analyses to assess the independent value of DRLR. Receiver operating characteristic curves (ROC) were calculated to evaluate the predictive efficacy of the signature in comparison to other clinicopathological characteristics in OC patients. Furthermore, we incorporated prognostic indicators including grade, age and risk score based on the package “regplot” to predict survival outcomes of OC patients at 1-, 3- and 5-years.

### Pathway and functional annotation analyses

We used Gene Ontology (GO) to gain insights into possible molecular mechanisms between low- and high-DRLRI groups via “clusterProfiler” R package. In addition, gene set enrichment analysis (GSEA) was carried out to distinguish primary pathways of action in the two risk sets, the cutoff value of which was |log2 fold change| > 1 together with false discovery rate (FDR) < 0.05.

### Description for immune landscapes and immune escape

We used “GSVA” and “GSEABase” R packages are used to estimate the abundance of tumor-infiltrating immune profiles and calculate differences in immune functions activity and immune cells infiltration between two risk statuses. The box plot visualized the differences in immune checkpoints between high- and low-risk groups. In addition, The Tumor Immune Dysfunction and Exclusion (TIDE) tool (http://tide.dfci.harvard.edu/) was employed to evaluate the possibility of tumor immune escape from immune surveillance in the gene expression profile of OC samples.

### Efficacy of chemotherapeutic agents

With the help of the pcakage “oncopredict”, developed for sensitive drugs prediction (27), we made efforts to seek specific medications for high-risk population. Specifically, the transcriptomics data of OC samples was fitted with drug sensitivity profiles of various cancer cell lines from the Genomics of Drug Sensitivity in Cancer (GDSC) database, the largest public resource to store the information for molecular indicators of drug response in tumor cell lines. Besides, an unpaired t-test was applied to compare the sensitivity of samples at different risk levels to multiple chemotherapeutic drugs.

## Results

### Recognition of hub lncRNAs

**Figure 1:**
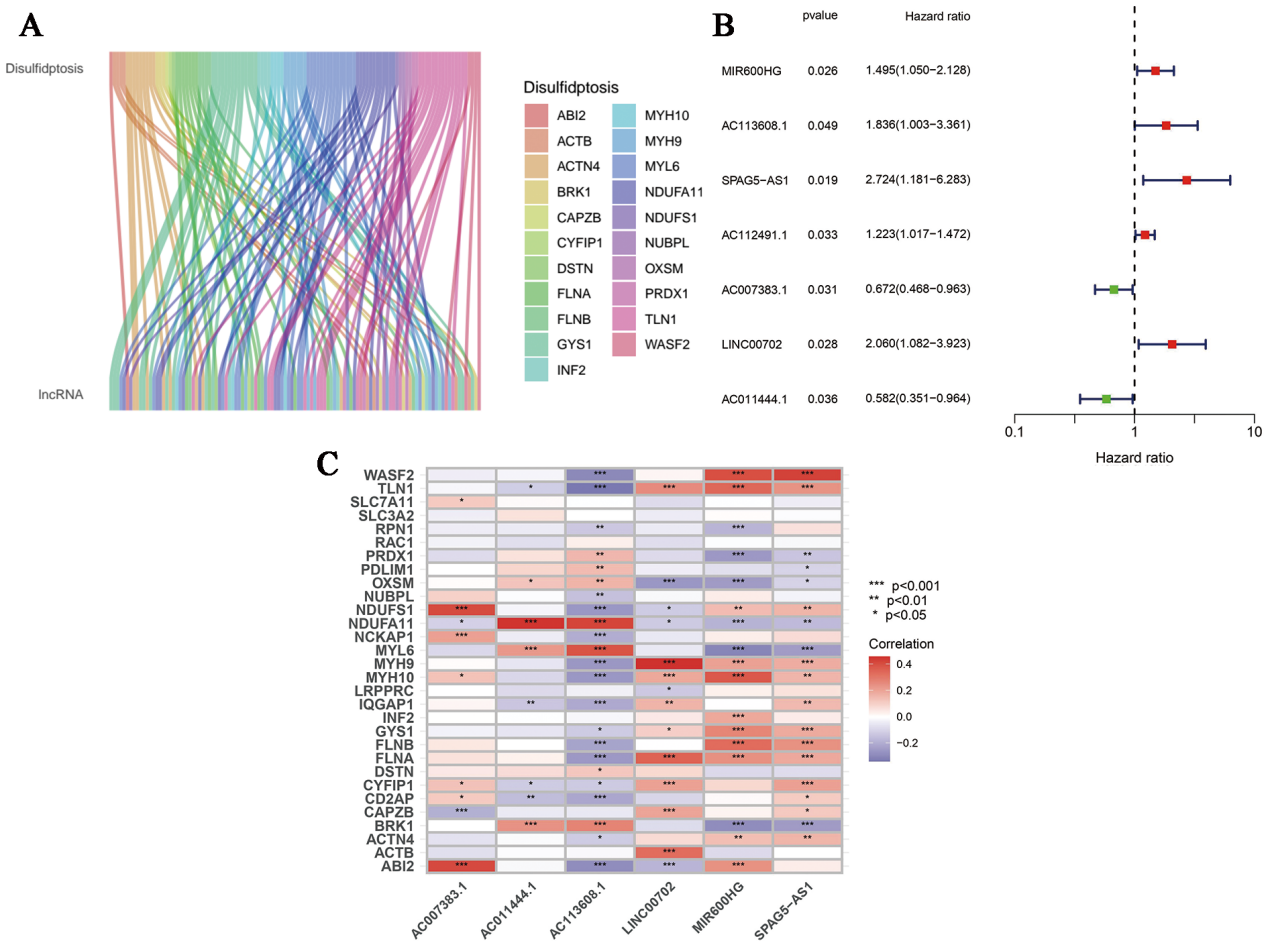
Generation of DRLRs in the model. **(A)** The Sankey diagram for all lncRNAs associated with disulfidptosis genes. **(B)** The forest plot for DRLRs with prognostic value. **(C)** The heat map showing correlations between disulfidptosis genes and core lncRNAs involved in the signature. Red represents positive correlation; Blue represents negative correlation.

First of all, we made a random internal classification of the TCGA-OV samples, which were divided into the experimental group and the verification group for follow-up analysis. Following the Spearman correlation analysis, 94 DRLRs were identified to be coexpressed with disulfidptosis-related genes with a correlation coefficient of 0.4 (**Figure 1A**), where nine lncRNAs were considered to hold prognostic value via univariate Cox analysis in the training group (**Figure 1B**). As displayed, six lncRNAs for the model construction were identified with the help of LASSO logistic regression by the following formula: risk score =* MIR600HG*exp * 0.507276626055331 + *AC113608.1*exp * 1.16817321415109 + *SPAG5-AS1*exp * 1.15825457491113 - *AC007383.1*exp * 0.303028968088069 + *LINC00702*exp * 0.702623955558811 - *AC011444.1*exp * 0.449171704758926 (**Table 1**). In addition, we drew a heat map to visualize the relevance between the six hub lncRNAs and disulfidptosis-associated genes (**Figure 1C**).

**Table 1:** The risk coefficient value of each lncRNA that makes up the signature.

LncRNA	Coef
MIR600HG	0.507276626055331
AC113608.1	1.16817321415109
SPAG5-AS1	1.15825457491113
AC007383.1	-0.303028968088069
LINC00702	0.702623955558811
AC011444.1	-0.449171704758926

LncRNA, long non-coding RNA; Coef, coefficient.

### Omnidirectional description and assessment of the model

**Figure 2:**
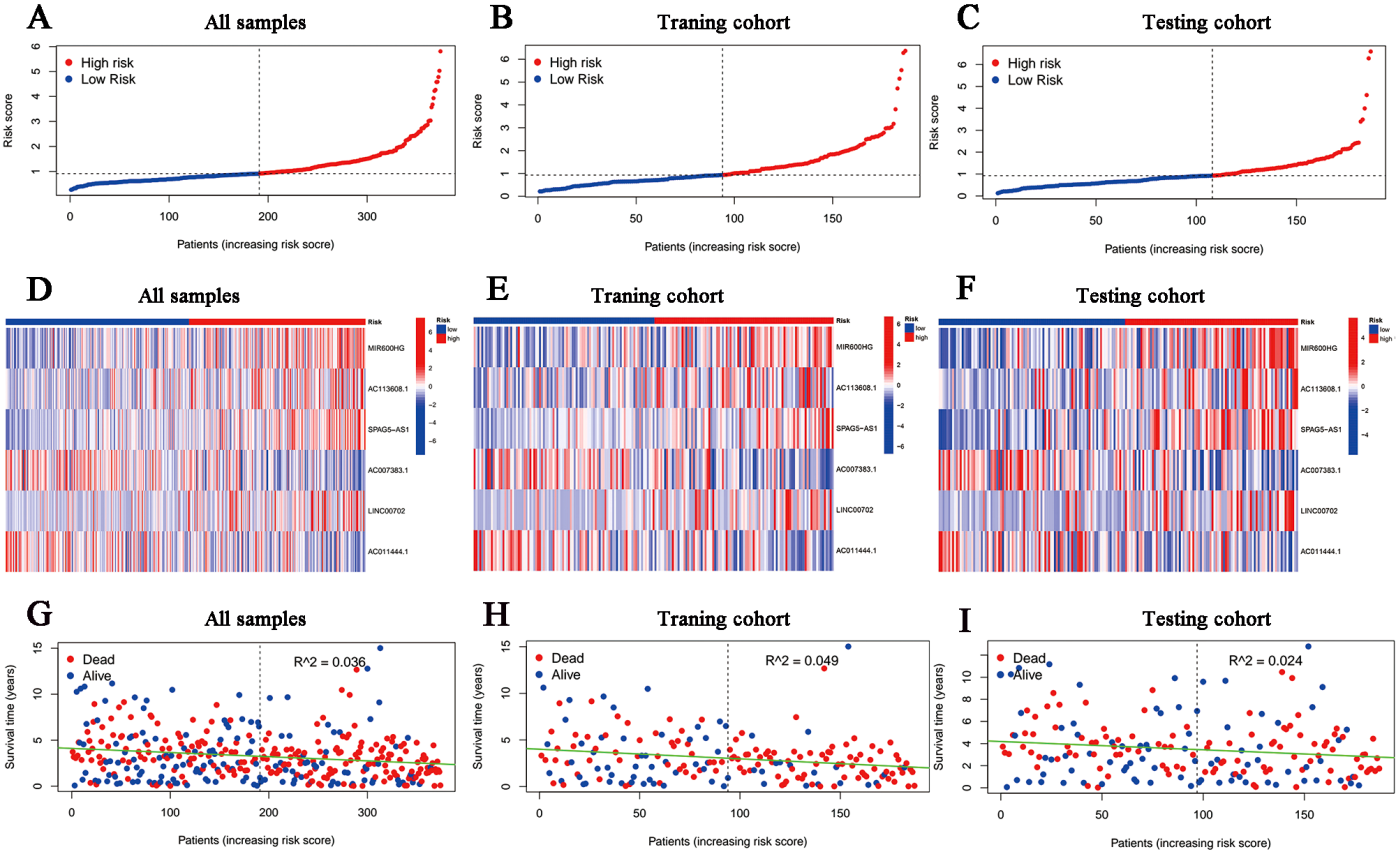
Overview of the risk stratification in the model. **(A-C)** The distributions of the risk scores in the entire, training and testing cohort, respectively. **(D-F)** Heat maps for the expression divergence of the six lncRNAs between different risk samples in the whole, training, and testing sets, respectively. **(G-I)** The relationship between survival status and risk scores in **(G)** the whole cohort, **(H)** the training cohort and **(I)** the testing cohort.

**Figure 3:**
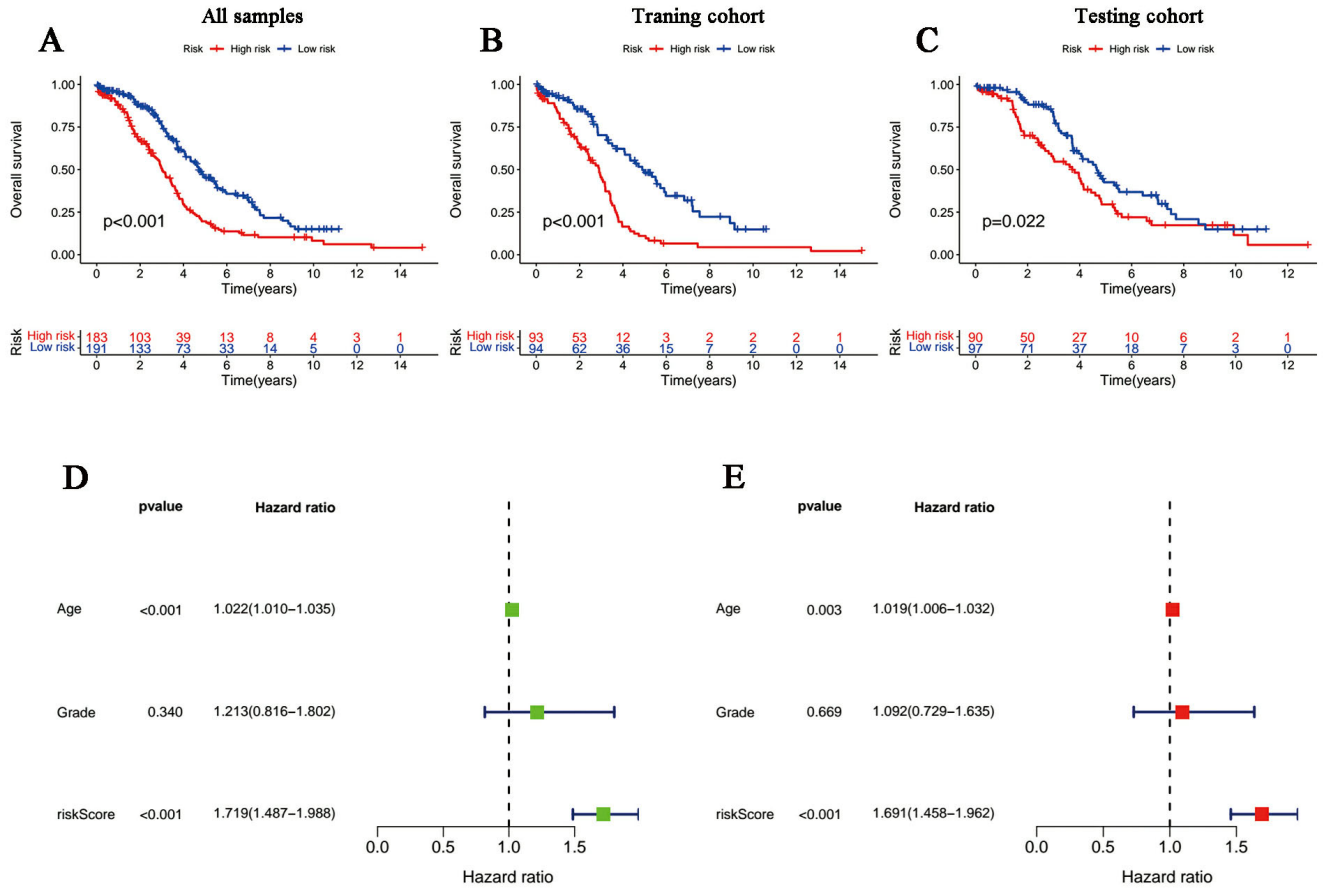
Survival curves and the Cox regression analysis of the signature. **(A-C)** Kaplan-Meier plots of low- and high-risk patients in **(A)** TCGA, **(B)** the training group and **(C)** the testing group. **(D, E)** Forest plots of **(D)** univariate Cox regression and **(E)** multivariate Cox regression.

According to the median risk score in the training cohort, we filtered out two risk statuses in different cohorts. **Figures 2A–C** show the distribution of patients sequentially according to different risk scores. Then, we presented the expression patterns of the six lncRNAs between two risk statuses from the whole set, training samples as well as testing samples, respectively (**Figures 2D–F**). Scattergrams showed the individual survival state of each patient in the entire, training and testing cohorts, which demonstrated that patients’ survival shortened with risk scores increasing (**Figures 2G–I**). Consistently, the Kaplan–Meier survival curve displayed that the high-risk patients performed dramatically poorer survival rates in comparison to those with low risk scores (**Figures 3A–C**). As expected, we observed a more favorable PFS in low-risk samples than in high-risk ones (**Supplementary Figure 1**).

**Figure 4:**
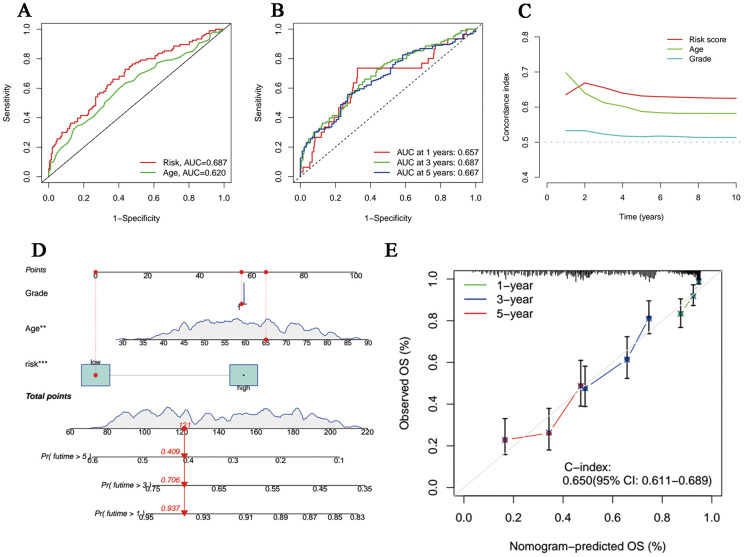
Evaluation and perfection of the prediction model. **(A)** Comparison between the risk score and the other clinicopathological variable in the accuracy of prognosis prediction. **(B)** Predicted survival rates on the basis of our signature at 1-, 3- and 5-year. **(C)** C-index curves of the risk score and other clinicopathological signatures. **(D)** The nomogram integrating grade, age and the risk score for survival outcomes prediction. **(E)** The calibration curve of the combination nomogram.

The univariate and multivariate Cox regression analyses incorporating tumor grade, age and risk score indicated that age and our signature are both independent prognostic factors for OC patients (**Figures 3D, E**). Furthermore, ROC curves (**Figure 4A**) demonstrated that the lncRNAs combination represented a more accurate item than the other clinicopathological indicator (area under curve = 0.687)). It was clear that the prediction power of 1-year, 3-year and 5-year survival rates were wonderful (**Figure 4B**). Similarly, the concordance index of our signature outperformed clinical characteristics of age and grade (**Figure 4C**). Subsequently, a nomogram integrating DRLR risk score and other clinical variables, including age and grade, was depicted to calculate the survival probabilities of OC patients at 1-year, 3-year and 5-year, respectively (**Figure 4D**). Finally, as shown in the calibration curve, the degree of fitness between the OS of observations and nomogram forecasts suggested that our work achieved satisfactory performance in patients with OC (**Figure 4E**).

**Figure 5:**
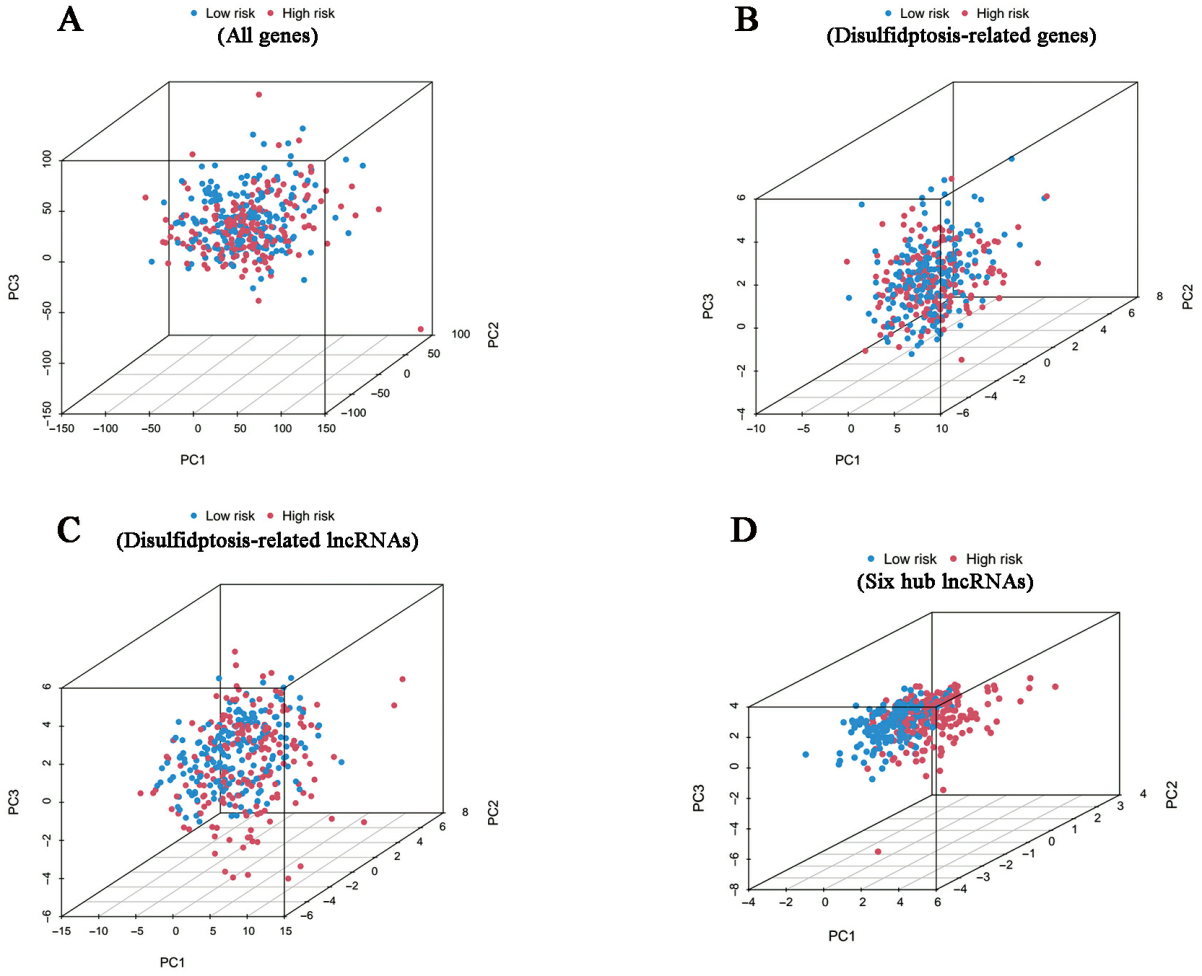
PCA results. Differences in distribution between the high- and low-risk groups in terms of all genes **(A)**, disulfidptosis-related genes **(B)**, disulfidptosis-related lncRNAs **(C)** and six hub lncRNAs **(D)**.

According to the investigation of spatial grouping performance, our signature discriminated the two risk clusters more brilliantly than all genes, disulfidptosis-associated genes and disulfidptosis-related lncRNAs (**Figure 5**).

### Functional enrichment analysis

**Figure 6:**
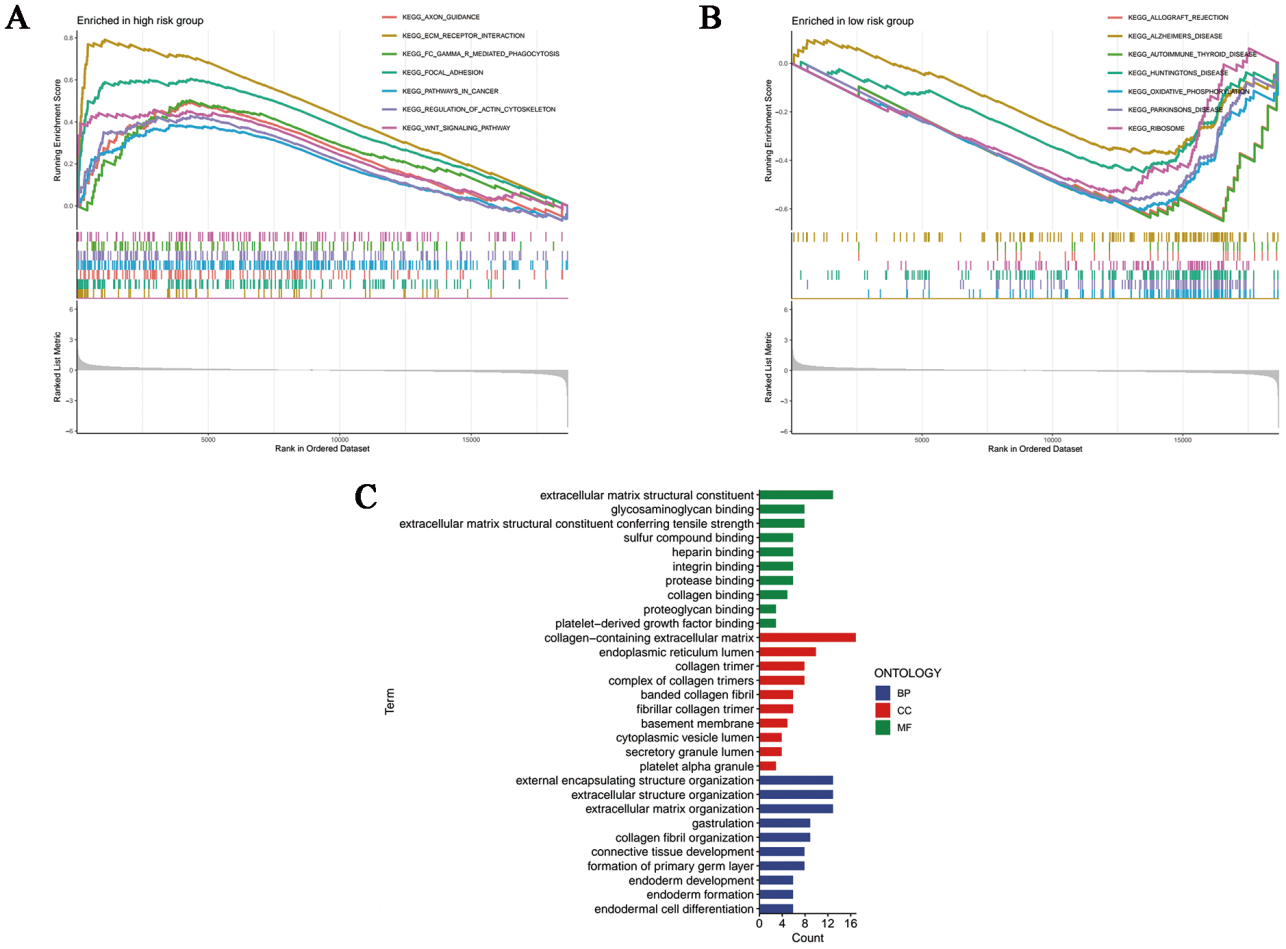
Functional enrichment analyses. GSEA analysis of the **(A)** high- and **(B)** low-risk samples. **(C)** Column plots of GO terms. BP, biological processes; CC, cellular components; MF, molecular functions.

Based on the Kyoto Encyclopedia of Genes and Genome gene sets, we first conducted GSEA analysis so as to shed light on the inner patterns of biological functions in high- and low-risk groups. As shown in **Figure 6A**, pathways in cancer and carcinogenic pathways such as ECM-receptor interaction, focal adhesion and WNT signaling pathway had elevated enrichment levels in the high-risk group, whereas the low-risk group was significantly enriched in the oxidative phosphorylation pathway, which was correlated with cellular energy metabolism (**Figure 6B**). Notably, according to the results of GO annotations, it was clear that differently expressed genes (DEGs) based on disulfidptosis were mainly involved in molecular functions (MF) associated with extracellular matrix structural constituent, glycosaminoglycan binding and extracellular matrix structural constituent conferring tensile strength. As for the cellular components (CC), they mainly functioned in collagen-containing extracellular matrix, endoplasmic reticulum lumen and collagen trimer. In addition, the biological processes (BP) were mainly related to the organization of external encapsulating structure, extracellular structure and extracellular matrix (**Figure 6C**).

### Assessment of immune infiltration and tumor microenvironment (TME)

**Figure 7:**
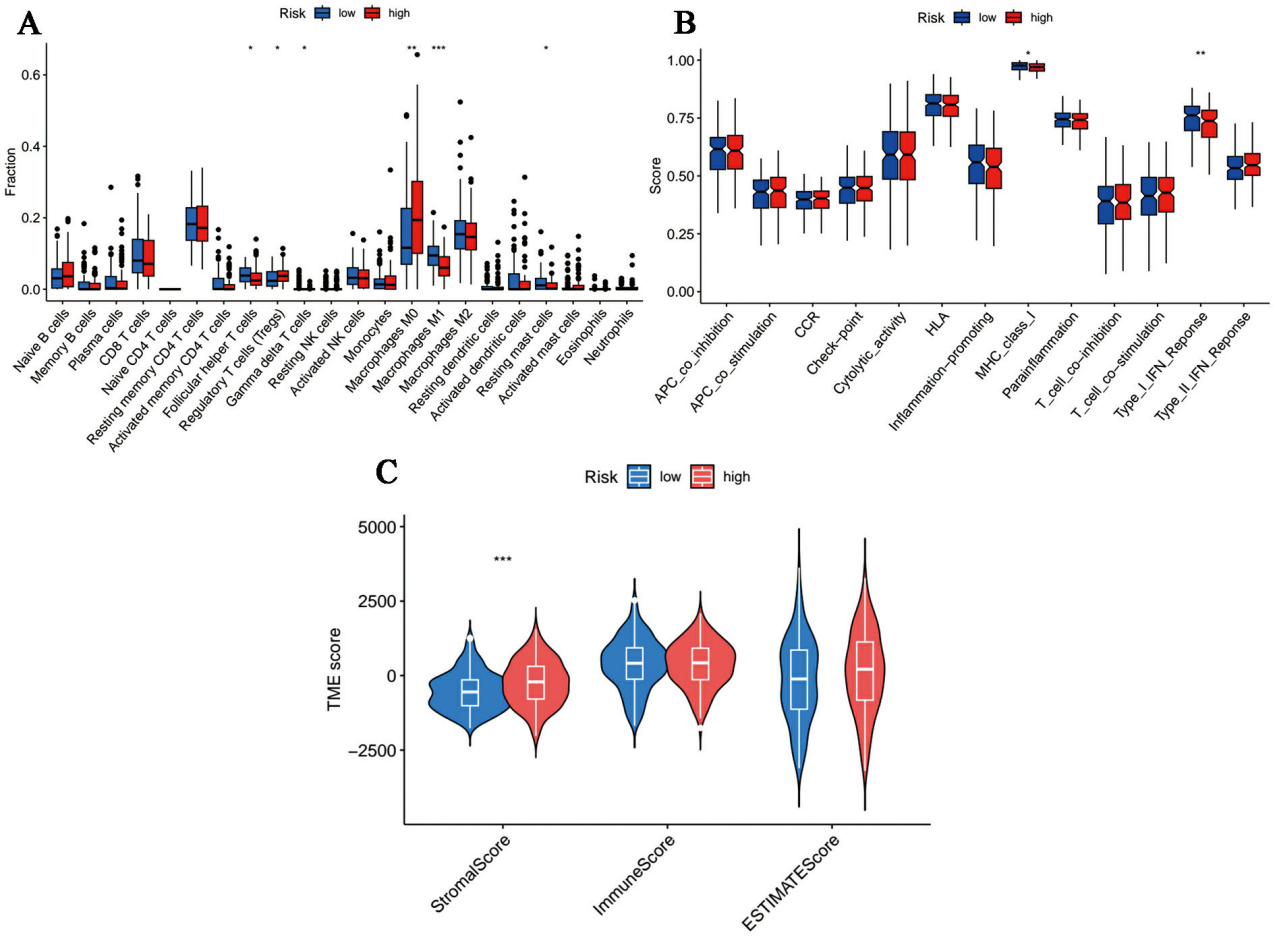
Immune microenvironment profiles in different risk samples. **(A, B)** The box plot of **(A)** immune cells and **(B)** immunological functions infiltration between different groups. **(C)** The stromal, immune and ESTIMATE scores in the low- and high-risk groups. (*p < 0.05, **p < 0.01, ***p < 0.001).

The overall dissimilarity of infiltrating immune cells is displayed in **Figures 7A, B** integrally. As presented, high-risk samples get more enrichment scores in regulatory T cells as well as M0 macrophages. And immunocytes, including T follicular helper cells, M1 Macrophages, gamma delta T cells and resting mast cells, are more enriched in the low-risk group. Besides, in the assessment of the immunologic functions, we noticed that high-risk OC patients were more likely to be associated with poorer immune systems compared to those in the low-risk category. Specifically, MHC_class_I and Type_I_IFN_Reponse are more active in the low-risk patients. Furthermore, OC tissue samples in the high-risk group had a significantly higher stromal score and ESTIMATE score in accordance with the quantification of matrix components, suggesting that there were stronger stromal components in the TME of these patients (**Figure 7C**).

### Predictions of immunotherapy response and chemotherapy drug sensitivity

**Figure 8:**
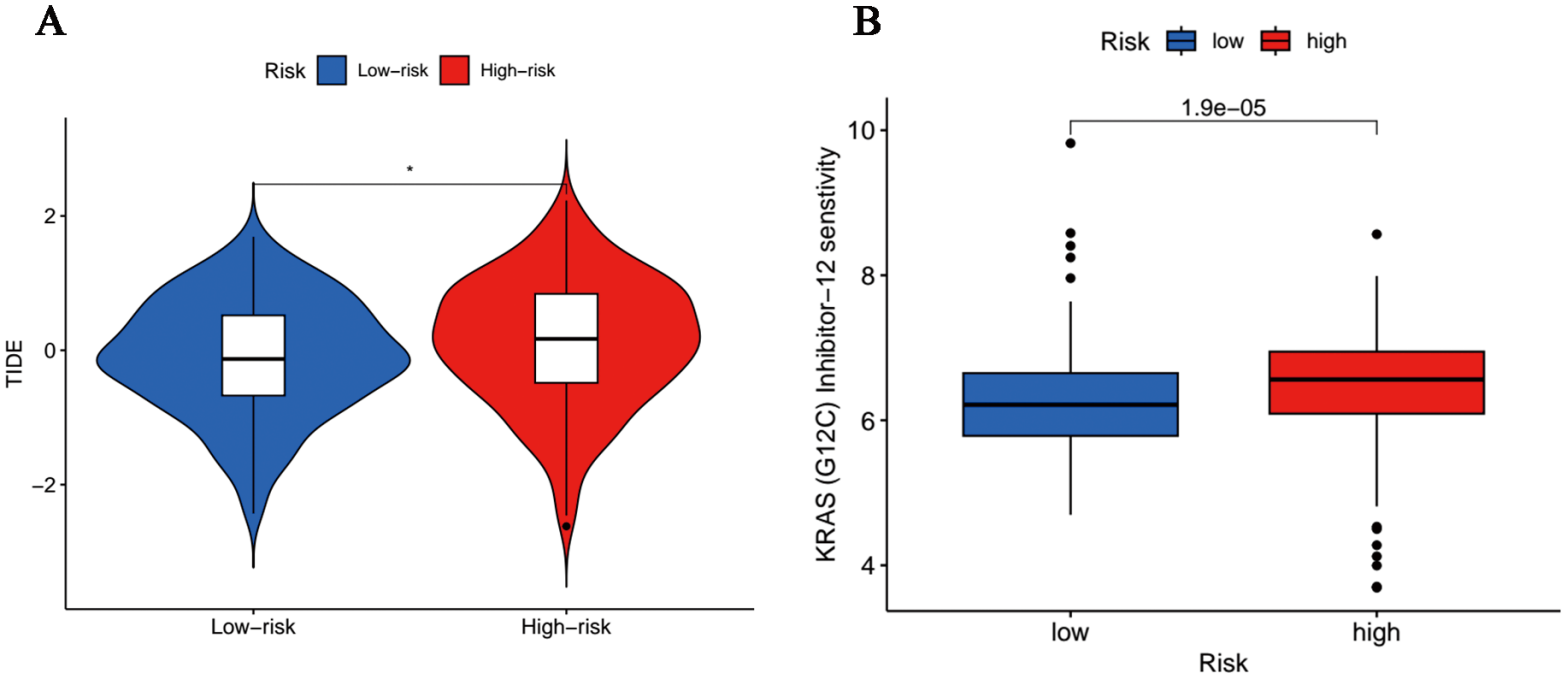
Tumor sensitivity to immunotherapy and chemotherapy. **(A)** Differences in immunoescape between low- and high-risk groups. (*p<0.05). **(B)** Sensitive chemotherapeutic agents for high-risk population.

Acting as immune system regulators, immune checkpoints play a vital role in the maintenance of self-immune tolerance and the regulation of peripheral tissue immune response, the continuous activation of which will bring about inhibited anti-tumor immunity and promoted tumorigenesis (28). In terms of immune checkpoints, including *PD-1* and *CTLA4*, we discovered that TIDE scores were more accumulated in the high-risk cohort (**Figure 8A**), which implied these patients hold potential immune dysfunction in tumors and could hardly benefit from immunotherapy. Moreover, it was revealed in the analysis of drug susceptibilities that commonly used chemotherapeutic drugs were more sensitive in low-risk people (**Supplementary Figure 2**), whereas those with high-risk scores responded better toward K-Ras (G12C) inhibitor-12, a K-Ras (G12C) inhibitor (**Figure 8B**).

## Discussion

Although disulfidptosis is a new form of cell death, which can make a difference to tumor initiation and progression, rapid efforts have been made to attempt to figure out the biological mechanisms underlying a variety of tumor types, including lung adenocarcinoma (29), adrenocortical carcinoma (30), cutaneous melanoma (31) and other gynecological tumors (13, 32). Recently, Cong et al. has investigated the potential of disulfidptosis in predicting the prognosis of OC patients (33). In comparaion, we have incorporated more updated disulfidptosis-related genes in this research. Meanwhile, our study on the prognosis of OC patients focuses on DRLRs, whose abnormal expression will account for invasive behaviors of cancerous cells and are identified as brilliant biomarkers for the diagnosis and treatment of cancers, with the advanced study of transcriptional regulation (34). Both contribute to making our study of disulfidptosis in the prognosis of ovarian cancer more comprehensive, which is notably absent in other research. Hitherto, DRLRs are still not idea and their significant prognostic values remain unknown territory in OC. In this article, we shared the achievements on the feasibility of DRLRs in the prediction of the prognosis in OC patients for the first time.

In the current study, we began by analyzing the expression levels of 30 DRGs and subsequently observed that they were significantly correlated with the expression of 94 lncRNAs (**Supplementary Table 2**). Subsequently, we adopted the univariate Cox analysis to acquire six prognosis-related lncRNAs, and followed by LASSO regression to obtain target lncRNAs for the model development in the train subgroup, namely *MIR600HG*, *AC113608.1*, *SPAG5-AS1*, *AC007383.1*, *LINC00702* and *AC011444.1*. Surprisingly, our model proved to present a fabulous performance in differentiating TME, immune infiltration and survival outcomes of OC patients. It was imperative that the implicative value of DRLRs in risk stratification of OC patients reached an absolute consensus in the test cohort. To recap, not only will our work pre-stratify the prognosis and treatment of OC patients, but it will also hopefully cause a sweeping change for the status quo of knowledge gaps about the disulfidptosis in OC.

We have performed a literature review on the six lncRNAs which contribute to the signature and noticed that four of them had been reported in several tumor types earlier. In particular, with elevated *AC011444.1* expression, metastatic osteosarcoma children were observed to experience poor survival outcomes (35), which was opposite to our results. As for *LINC00702*, *in vitro* trials have demonstrated that it could enhance the progression of malignant meningioma by up-regulating Wnt/β-catenin pathway on the one hand (36). Likewise, the malignant behaviors of breast cancer cells seem to be facilitated when it comes to highly expressed *LINC00702 (* 37). On the other hand, overexpressed *LINC00702* leads to inhibited proliferation of bladder cancer cells by targeting *DUSP1 in vitro (* 38). Herein, we discovered that *LINC00702* represented a significant risk factor for survival in clinical association analysis, and would cause poor prognostic features in high-risk OC patients. Collectively, *LINC00702* may play a dual role in cancer pathogenesis acting as a tumor suppressor or tumor-promoting factor, which is attributed to the heterogeneity of tumors. Then, it has been reported that the malignant characteristics of tumor cells could be inhibited along with *MIR600HG* elevation in pancreatic cancer (39). On the contrary, a recent study has suggested that *MIR600GH* was an oncogene in colorectal cancer, the high expression of which promoted tumor invasiveness and induced advanced TNM stage (40). Notably, the only trial involving *MIR600GH* in OC showed its adverse effect on survival outcomes (41), which was consistent with our findings that increased *MIR600HG* resulted in poor prognosis of OC patients. One previous study has provided the initial insight into autophagy and apoptosis associated with *SPAG5-AS1* in podocytes (42), which was first illustrated as a regulator of disulfidptosis in the current investigation. Crucially, to the best of our knowledge, endeavors have so far failed to investigate the role of *AC007383.1* and *AC113608.1* in any diseases, and this is not only the first attempt to apply them to characterize ranked risk scores for OC patients, but also the first exploration in all cancer types. In a nutshell, overexpression of the six lncRNAs in our signature was relevant to poor survival outcomes in OC patients.

The remodeling of extracellular matrix is related to the establishment of an immunosuppressive environment in invasive cancers, including OC (43). Regarding the functional analysis, we noticed that DEGs were highly closed to the extracellular matrix more than cancer-related pathways. More specifically, GSEA results revealed that ECM-receptor interaction was significantly enriched in high-risk patients. Furthermore, the differences between the two risk statuses of MF, BP as well as CC were intimately coupled with the extracellular matrix, which were visualized in the GO bar plot. This was also supported by the assessment of stromal content, which showed that high-risk cases had higher stromal scores. This suggests that disulfidptosis leading to the change of extracellular matrix components appears to be a rational speculation, which will be gradually revealed along with the in-depth exploration. Principally, from the results of functional enrichment analysis, we noticed significant differences in the two core pathways of disulfidptosis, including regulation of actin cytoskeleton and cell adhesion, indicating that the activity of disulfidptosis was significantly different between the two risk samples.

The immune environment, one of the hotspots of the TME, has proven to profoundly influence the occurrence and migration/invasion of tumors cells (44). According to our results, it was found that the amount of regulatory T cells and M0 macrophages were significantly increased in the high-risk population, while the abundance of M1 phenotype macrophages decreased in those ones. It seems that the polarization process of naive macrophages tends to be suppressed in the high-risk set. Furthermore, immune cells with antitumor properties, like T follicular helper cells, gamma delta T cells and resting mast cells were all more densely-infiltrated in the tumor immune microenvironment of low-risk patients. Currently, there is an inevitable fact that regulatory T cells particularly contribute to suppressing T cell activation in TME (45). In addition, it is thought that increased frequencies of T follicular helper cells are related to a better prognosis in solid organ tumors (46). M1 macrophages were demonstrated to possess tumor-resistant effects through intrinsic phagocytosis and enhanced antitumor inflammation (47). In the tumor microenvironment (TME), gamma delta T cells account for recognizing and destroying infected or transformed cells to prevent malignancy formation (48). Recently, gamma delta T cells have been reported to exhibit more severe functional exhaustion than NK or CD8(+) T cells in colorectal cancer (49). In the previous study, we noted the potential relationship between impaired major histocompatibility complex (MHC) class I functions and poor prognosis of OC (50), which was further supported by the current observation. Losing MHC class I molecules, cancer cells can escape from T cells-mediated tumor killing (51). Moreover, the robust antitumor immune functions of Type I IFN Reponses have also been identified in the low-risk cohort (52). Notably, low-risk OC patients hold lower TIDE scores in the present study, indicating that these patients are more likely to respond well to immune checkpoint inhibitors than those with high risk scores. On the whole, we found that patients in the high-risk cohort had enormously poorer antitumor immune responses and stronger immune evasion capacity, that is, low-risk patients were better suited to receive immunotherapy.

Thereafter, in the prediction of chemosensitivity, we demonstrated that the most commonly used chemotherapeutic agents, Alpelisib, Cediranib and Mitoxantrone usually result in limited therapeutic effect for high-risk patients, which may be responsible for the early emergence of chemoresistance in certain patients. Admittedly, we identified K-Ras (G12C) inhibitor-12 as an effective chemodrug targeted for high-risk patients, which may help address the immediate needs of prolonging the survival of high-risk OC population. K-Ras (G12C) inhibitor-12, an oncogenic K-Ras(G12C) inhibitor, which was essential to sustain T cell infiltration in reaction to immune checkpoint inhibitor in a pro-inflammatory manner, has provided benefits for cancer patients (53). However, there is no definite application record about K-Ras (G12C) inhibitor-12 in OC until very recently, so our study may broaden new opportunities for therapy strategies among high-risk population.

## Conclusion

In summary, we defined a pioneering signature based on six lncRNAs related to disulfidptosis in OC and assessed using internal validation. Our model provided a brilliant application for predicting the survival outcomes and immune profiles of OC patients with independence, specificity and accuracy. More importantly, it could be proposed as a novel biomarker for immunotherapy sensitivity and chemosensitivity, as well as a guide for options of suitable treatment in OC. Typically, internal validation is not sufficient to fully validate a model, for which external validation and further experimental programs are still needed.

## Data Availability

The datasets presented in this study can be found in online repositories. The names of the repository/repositories and accession number(s) can be found below: https://portal.gdc.cancer.gov/, TCGA-OV; https://xenabrowser.net/.

## Supplementary material

Supplementary Figure 1 Comparison of PFS between two risk groups based on the signature.

Supplementary Figure 2 Commonly used chemotherapeutic drugs with high sensitivity to low-risk population. **(A)** Alpelisib; **(B)** Cediranib; **(C)** Mitoxantrone.

## References

[1] Block MS Dietz AB Gustafson MP Kalli KR Erskine CL Youssef B. Th17-inducing autologous dendritic cell vaccination promotes antigen-specific cellular and humoral immunity in ovarian cancer patients. Nat Commun. (2020) 11:5173. doi: 10.1038/s41467-020-18962-z 33057068 PMC7560895

[2] Sung H Ferlay J Siegel RL Laversanne M Soerjomataram I Jemal A. Global cancer statistics 2020: GLOBOCAN estimates of incidence and mortality worldwide for 36 cancers in 185 countries. CA Cancer J Clin. (2021) 71:209–49. doi: 10.3322/caac.21660 33538338

[3] González-Martín A Pothuri B Vergote I Graybill W Lorusso D McCormick CC. Progression-free survival and safety at 3.5years of follow-up: results from the randomised phase 3 PRIMA/ENGOT-OV26/GOG-3012 trial of niraparib maintenance treatment in patients with newly diagnosed ovarian cancer. Eur J Cancer. (2023) 189:112908. doi: 10.1016/j.ejca.2023.04.024 37263896

[4] Siegel RL Miller KD Wagle NS Jemal A. Cancer statistics, 2023. CA Cancer J Clin. (2023) 73:17–48. doi: 10.3322/caac.21763 36633525

[5] Liu X Nie L Zhang Y Yan Y Wang C Colic M. Actin cytoskeleton vulnerability to disulfide stress mediates disulfidptosis. Nat Cell Biol. (2023) 25:404–14. doi: 10.1038/s41556-023-01091-2 PMC10027392 36747082

[6] Jiang L Kon N Li T Wang SJ Su T Hibshoosh H. Ferroptosis as a p53-mediated activity during tumour suppression. Nature. (2015) 520:57–62. doi: 10.1038/nature14344 25799988 PMC4455927

[7] Fantone S Piani F Olivieri F Rippo MR Sirico A Di Simone N. Role of SLC7A11/xCT in ovarian cancer. Int J Mol Sci. (2024) 25. doi: 10.3390/ijms25010587 PMC10779187 38203758

[8] Zhang X Zheng X Ying X Xie W Yin Y Wang X. CEBPG suppresses ferroptosis through transcriptional control of SLC7A11 in ovarian cancer. J Transl Med. (2023) 21:334. doi: 10.1186/s12967-023-04136-0 37210575 PMC10199564

[9] Chen B Dragomir MP Yang C Li Q Horst D Calin GA. Targeting non-coding RNAs to overcome cancer therapy resistance. Signal Transduct Target Ther. (2022) 7:121. doi: 10.1038/s41392-022-00975-3 35418578 PMC9008121

[10] Dragomir MP Manyam GC Ott LF Berland L Knutsen E Ivan C. FuncPEP: A database of functional peptides encoded by non-coding RNAs. Noncoding RNA. (2020) 6. doi: 10.3390/ncrna6040041 PMC7712257 32977531

[11] Liu S Zheng Y Li S Du Y Liu X Tang H. Integrative landscape analysis of prognostic model biomarkers and immunogenomics of disulfidptosis-related genes in breast cancer based on LASSO and WGCNA analyses. J Cancer Res Clin Oncol. (2023). doi: 10.1007/s00432-023-05372-z PMC10645620 37736788

[12] Xia Q Yan Q Wang Z Huang Q Zheng X Shen J. Disulfidptosis-associated lncRNAs predict breast cancer subtypes. Sci Rep. (2023) 13:16268. doi: 10.1038/s41598-023-43414-1 37758759 PMC10533517

[13] Liu L Liu J Lyu Q Huang J Chen Y Feng C. Disulfidptosis-associated LncRNAs index predicts prognosis and chemotherapy drugs sensitivity in cervical cancer. Sci Rep. (2023) 13:12470. doi: 10.1038/s41598-023-39669-3 37528124 PMC10394072

[14] Xing F Qin Y Xu J Wang W Zhang B. Construction of a novel disulfidptosis-related lncRNA prognostic signature in pancreatic cancer. Mol Biotechnol. (2023). doi: 10.1007/s12033-023-00875-z 37733182

[15] Dong X Liao P Liu X Yang Z Wang Y Zhong W. Construction and validation of a reliable disulfidptosis-related lncRNAs signature of the subtype, prognostic, and immune landscape in colon cancer. Int J Mol Sci. (2023) 24. doi: 10.3390/ijms241612915 PMC10454603 37629096

[16] Xue W Qiu K Dong B Guo D Fu J Zhu C. Disulfidptosis-associated long non-coding RNA signature predicts the prognosis, tumor microenvironment, and immunotherapy and chemotherapy options in colon adenocarcinoma. Cancer Cell Int. (2023) 23:218. doi: 10.1186/s12935-023-03065-8 37759294 PMC10523716

[17] Yang Z Cao S Wang F Du K Hu F. Characterization and Prognosis of Biological Microenvironment in Lung Adenocarcinoma through a Disulfidptosis-Related lncRNAs Signature. Genet Res (Camb). (2023) 2023:6670514. doi: 10.1155/2023/6670514 37575978 PMC10421709

[18] Zhang HB Pan JY Zhu T. A disulfidptosis-related lncRNA prognostic model to predict survival and response to immunotherapy in lung adenocarcinoma. Front Pharmacol. (2023) 14:1254119. doi: 10.3389/fphar.2023.1254119 37822882 PMC10563764

[19] Chen Y Jin C Cui J Diao Y Wang R Xu R. Single-cell sequencing and bulk RNA data reveal the tumor microenvironment infiltration characteristics of disulfidptosis related genes in breast cancer. J Cancer Res Clin Oncol. (2023) 149:12145–64. doi: 10.1007/s00432-023-05109-y PMC11796854 37428249

[20] Feng Z Zhao Q Ding Y Xu Y Sun X Chen Q. Identification a unique disulfidptosis classification regarding prognosis and immune landscapes in thyroid carcinoma and providing therapeutic strategies. J Cancer Res Clin Oncol. (2023) 149:11157–70. doi: 10.1007/s00432-023-05006-4 PMC11796729 37347261

[21] Li Y Tang M Dang W Zhu S Wang Y. Identification of disulfidptosis-related subtypes, characterization of tumor microenvironment infiltration, and development of a prognosis model in colorectal cancer. J Cancer Res Clin Oncol. (2023) 149:13995–4014. doi: 10.1007/s00432-023-05211-1 PMC11796609 37543978

[22] Qi C Ma J Sun J Wu X Ding J. The role of molecular subtypes and immune infiltration characteristics based on disulfidptosis-associated genes in lung adenocarcinoma. Aging (Albany NY). (2023) 15:5075–95. doi: 10.18632/aging.204782 PMC10292876 37315289

[23] Wang T Guo K Zhang D Wang H Yin J Cui H. Disulfidptosis classification of hepatocellular carcinoma reveals correlation with clinical prognosis and immune profile. Int Immunopharmacol. (2023) 120:110368. doi: 10.1016/j.intimp.2023.110368 37247499

[24] Xu K Zhang Y Yan Z Wang Y Li Y Qiu Q. Identification of disulfidptosis related subtypes, characterization of tumor microenvironment infiltration, and development of DRG prognostic prediction model in RCC, in which MSH3 is a key gene during disulfidptosis. Front Immunol. (2023) 14:1205250. doi: 10.3389/fimmu.2023.1205250 37426643 PMC10327482

[25] Xu L Wang S Zhang D Wu Y Shan J Zhu H. Machine learning- and WGCNA-mediated double analysis based on genes associated with disulfidptosis, cuproptosis and ferroptosis for the construction and validation of the prognostic model for breast cancer. J Cancer Res Clin Oncol. (2023) 149:16511–23. doi: 10.1007/s00432-023-05378-7 PMC11798189 37712959

[26] Yang L Liu J Li S Liu X Zheng F Xu S. Based on disulfidptosis, revealing the prognostic and immunological characteristics of renal cell carcinoma with tumor thrombus of vena cava and identifying potential therapeutic target AJAP1. J Cancer Res Clin Oncol. (2023) 149:9787–804. doi: 10.1007/s00432-023-04877-x PMC11796798 37247081

[27] Maeser D Gruener RF Huang RS. oncoPredict: an R package for predicting *in vivo* or cancer patient drug response and biomarkers from cell line screening data. Brief Bioinform. (2021) 22. doi: 10.1093/bib/bbab260 PMC8574972 34260682

[28] Muaibati M Abuduyilimu A Zhang T Dai Y Li R Huang F. Efficacy of immune checkpoint inhibitor monotherapy or combined with other small molecule-targeted agents in ovarian cancer. Expert Rev Mol Med. (2023) 25:e6. doi: 10.1017/erm.2023.3 36691778

[29] Ma X Deng Z Li Z Ma T Li G Zhang C. Leveraging a disulfidptosis/ferroptosis-based signature to predict the prognosis of lung adenocarcinoma. Cancer Cell Int. (2023) 23:267. doi: 10.1186/s12935-023-03125-z 37946181 PMC10634118

[30] Liu T Ren Y Wang Q Wang Y Li Z Sun W. Exploring the role of the disulfidptosis-related gene SLC7A11 in adrenocortical carcinoma: implications for prognosis, immune infiltration, and therapeutic strategies. Cancer Cell Int. (2023) 23:259. doi: 10.1186/s12935-023-03091-6 37919768 PMC10623781

[31] Zhao Y Wei Y Fan L Nie Y Li J Zeng R. Leveraging a disulfidptosis-related signature to predict the prognosis and immunotherapy effectiveness of cutaneous melanoma based on machine learning. Mol Med. (2023) 29:145. doi: 10.1186/s10020-023-00739-x 37884883 PMC10601311

[32] Shi S Tang X Liu H. Disulfidptosis-related lncRNA for the establishment of novel prognostic signature and therapeutic response prediction to endometrial cancer. Reprod Sci. (2023). doi: 10.1007/s43032-023-01382-x 37880552

[33] Cong Y Cai G Ding C Zhang H Chen J Luo S. Disulfidptosis-related signature elucidates the prognostic, immunologic, and therapeutic characteristics in ovarian cancer. Front Genet. (2024) 15:1378907. doi: 10.3389/fgene.2024.1378907 38694875 PMC11061395

[34] Chi Y Wang D Wang J Yu W Yang J. Long non-coding RNA in the pathogenesis of cancers. Cells. (2019) 8. doi: 10.3390/cells8091015 PMC6770362 31480503

[35] Wei J Fang DL Huang CK Hua SL Lu XS. Screening a novel signature and predicting the immune landscape of metastatic osteosarcoma in children via immune-related lncRNAs. Transl Pediatr. (2021) 10:1851–66. doi: 10.21037/tp PMC8349967 34430433

[36] Li T Ren J Ma J Wu J Zhang R Yuan H. LINC00702/miR-4652-3p/ZEB1 axis promotes the progression of Malignant meningioma through activating Wnt/β-catenin pathway. BioMed Pharmacother. (2019) 113:108718. doi: 10.1016/j.biopha.2019.108718 30849635

[37] Chai D Yang C Liu Y Li H Lian B Bai Z. Knockdown of LINC00702 inhibits the growth and induces apoptosis of breast cancer through the Wnt/β-catenin pathway. Heliyon. (2023) 9:e20651. doi: 10.1016/j.heliyon.2023.e20651 37860544 PMC10582296

[38] Pan W Han J Wei N Wu H Wang Y Sun J. LINC00702-mediated DUSP1 transcription in the prevention of bladder cancer progression: Implications in cancer cell proliferation and tumor inflammatory microenvironment. Genomics. (2022) 114:110428. doi: 10.1016/j.ygeno.2022.110428 35809838

[39] Chen F Zheng X Liang W Jiang C Su D Fu B. Long noncoding RNA MIR600HG binds to microRNA-125a-5p to prevent pancreatic cancer progression via mitochondrial tumor suppressor 1-dependent suppression of extracellular regulated protein kinases signaling pathway. Pancreas. (2022) 51:1434–43. doi: 10.1097/MPA.0000000000002185 37099789

[40] Huang Y Wang L Liu D. lncRNA MIR600HG induces the proliferation and invasion of colorectal cancer cells via regulating miR-144-3p/KIF3A. Int Immunopharmacol. (2022) 108:108686. doi: 10.1016/j.intimp.2022.108686 35378445

[41] Cao X Zhang Q Zhu Y Huo X Bao J Su M. Derivation, comprehensive analysis, and assay validation of a pyroptosis-related lncRNA prognostic signature in patients with ovarian cancer. Front Oncol. (2022) 12:780950. doi: 10.3389/fonc.2022.780950 35280739 PMC8912994

[42] Xu J Deng Y Wang Y Sun X Chen S Fu G. SPAG5-AS1 inhibited autophagy and aggravated apoptosis of podocytes via SPAG5/AKT/mTOR pathway. Cell Prolif. (2020) 53:e12738. doi: 10.1111/cpr.12738 31957155 PMC7046304

[43] Pearce O Delaine-Smith RM Maniati E Nichols S Wang J Böhm S. Deconstruction of a metastatic tumor microenvironment reveals a common matrix response in human cancers. Cancer Discovery. (2018) 8:304–19. doi: 10.1158/2159-8290.CD-17-0284 PMC5837004 29196464

[44] Fane M Weeraratna AT. How the ageing microenvironment influences tumour progression. Nat Rev Cancer. (2020) 20:89–106. doi: 10.1038/s41568-019-0222-9 31836838 PMC7377404

[45] Shan F Somasundaram A Bruno TC Workman CJ Vignali D. Therapeutic targeting of regulatory T cells in cancer. Trends Cancer. (2022) 8:944–61. doi: 10.1016/j.trecan.2022.06.008 PMC9588644 35853825

[46] Gutiérrez-Melo N Baumjohann D. T follicular helper cells in cancer. Trends Cancer. (2023) 9:309–25. doi: 10.1016/j.trecan.2022.12.007 36642575

[47] Liu J Geng X Hou J Wu G. New insights into M1/M2 macrophages: key modulators in cancer progression. Cancer Cell Int. (2021) 21:389. doi: 10.1186/s12935-021-02089-2 34289846 PMC8296555

[48] Saura-Esteller J de Jong M King LA Ensing E Winograd B de Gruijl TD. Gamma delta T-cell based cancer immunotherapy: past-present-future. Front Immunol. (2022) 13:915837. doi: 10.3389/fimmu.2022.915837 35784326 PMC9245381

[49] Ouyang Y Yu M Liu T Suo M Qiao J Wang L. An activated dendritic-cell-related gene signature indicative of disease prognosis and chemotherapy and immunotherapy response in colon cancer patients. Int J Mol Sci. (2023) 24. doi: 10.3390/ijms242115959 PMC10647347 37958942

[50] Liu Y Liu S Yan L Zhang Q Liu W Huang X. Contribution of m5C RNA modification-related genes to prognosis and immunotherapy prediction in patients with ovarian cancer. Mediators Inflammation. (2023) 2023:1400267. doi: 10.1155/2023/1400267 PMC10661868 38022687

[51] Garrido F Aptsiauri N. Cancer immune escape: MHC expression in primary tumours versus metastases. Immunology. (2019) 158:255–66. doi: 10.1111/imm.13114 PMC6856929 31509607

[52] Fenton SE Saleiro D Platanias LC. Type I and II interferons in the anti-tumor immune response. Cancers (Basel). (2021) 13. doi: 10.3390/cancers13051037 PMC7957896 33801234

[53] Liu J Kang R Tang D. The KRAS-G12C inhibitor: activity and resistance. Cancer Gene Ther. (2022) 29:875–8. doi: 10.1038/s41417-021-00383-9 34471232

