# Supplementary figures and images for: Comprehensive identification of a disulfidptosis-associated long non-coding RNA signature to predict the prognosis and treatment options in ovarian cancer
Source: Front Endocrinol (Lausanne). 2024 Sep 13;15:1434705. doi: 10.3389/fendo.2024.1434705 (PMC11427372; doi:10.3389/fendo.2024.1434705)

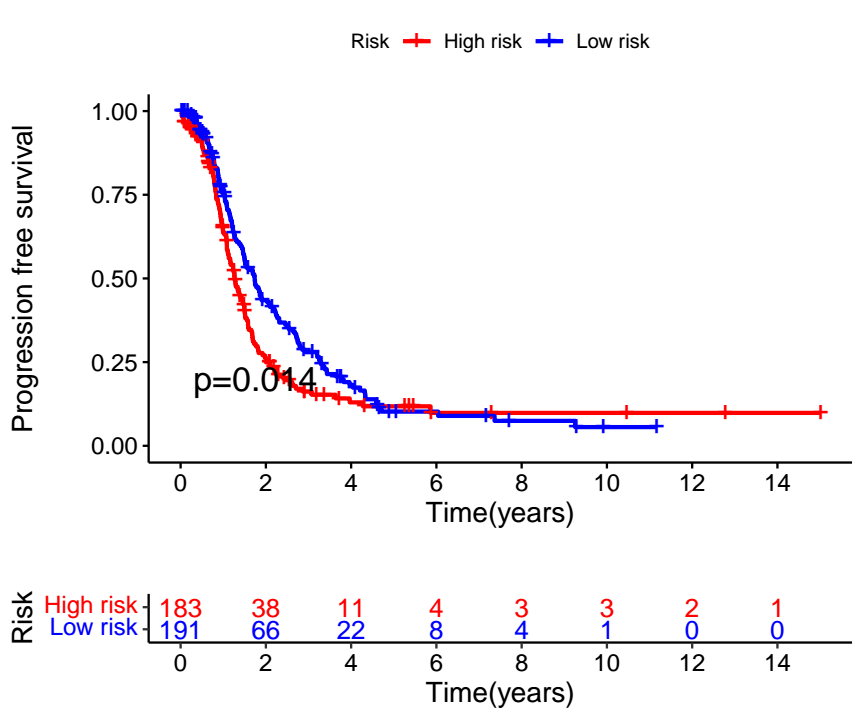

Supplement: Supplementary Figure 1 — Comparison of PFS between two risk groups based on the signature. [file Image1.tif]

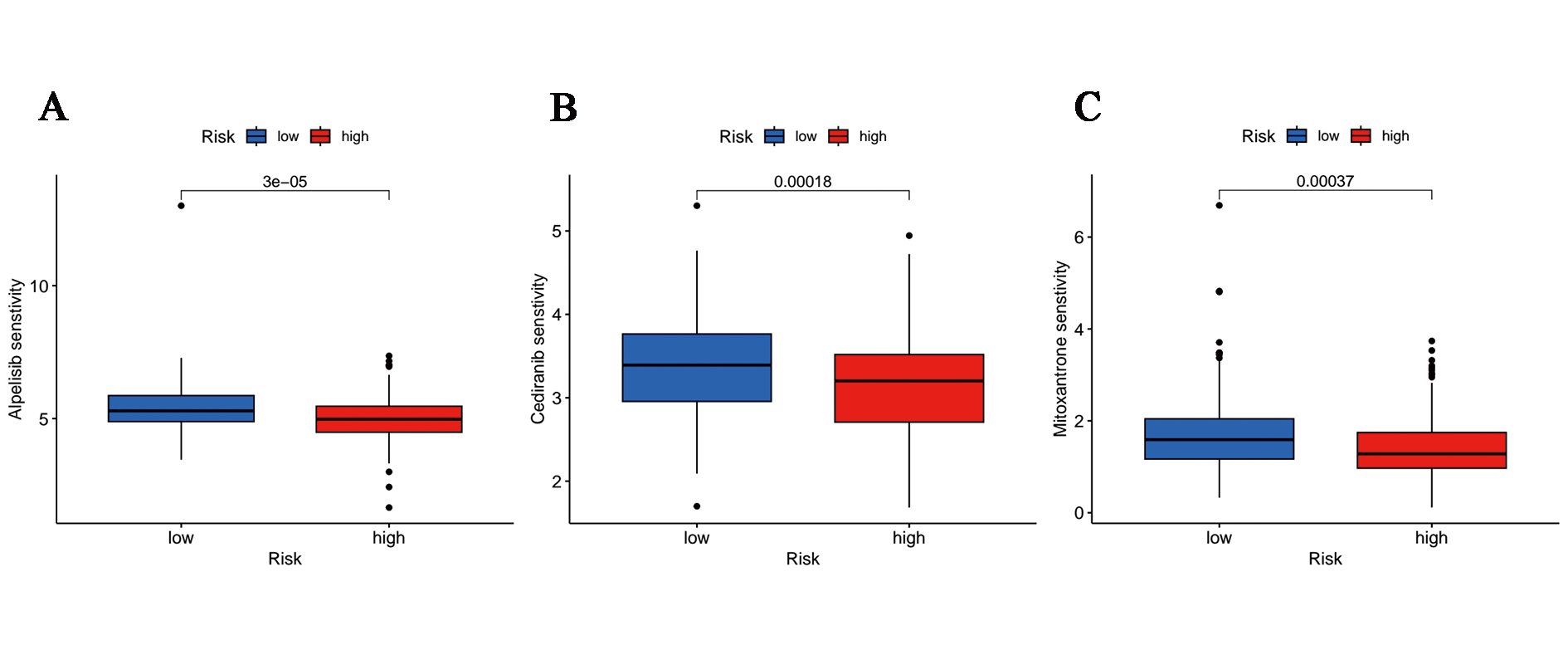

Supplement: Supplementary Figure 2 — Commonly used chemotherapeutic drugs with high sensitivity to low-risk population. (A) Alpelisib; (B) Cediranib; (C) Mitoxantrone. [file Image2.tif]
